# The PE/PPE family proteins of *Mycobacterium tuberculosis*: evolution, function, and prospects for tuberculosis control

**DOI:** 10.3389/fimmu.2025.1606311

**Published:** 2025-06-17

**Authors:** Zhijing Zhang, Le Dong, Xin Li, Taibing Deng, Qinglan Wang

**Affiliations:** ^1^ Institute of Respiratory Health, Frontiers Science Center for Disease-related Molecular Network, West China Hospital, Sichuan University, Chengdu, China; ^2^ West China School of Basic Medical Sciences & Forensic Medicine, Sichuan University, Chengdu, Sichuan, China; ^3^ College of Life Sciences, Sichuan University, Chengdu, Sichuan, China; ^4^ School of Clinical Medicine, North Sichuan Medical College, Nanchong, Sichuan, China

**Keywords:** Mycobacterium tuberculosis, PE/PPE, evolution, outer membrane, porin, vaccine

## Abstract

Tuberculosis (TB), caused by *Mycobacterium tuberculosis* (Mtb), remains a leading global health threat, exacerbated by drug resistance and inadequate vaccine efficacy. The PE/PPE protein family, unique to mycobacteria, constitutes ~10% of the Mtb genome and plays critical roles in bacterial physiology, immune evasion, and host-pathogen interactions. This review synthesizes advances in understanding the evolutionary expansion, structural diversity, and functional versatility of PE/PPE proteins, emphasizing their co-evolution with type VII secretion systems (T7SS). We highlight their roles in nutrient acquisition, immune modulation, and pathogenesis, alongside their potential as diagnostic and vaccine targets. Clinical progress in PE/PPE-based vaccines, such as M72/AS01E and ID93/GLA-SE, underscores their promise in combating TB, while challenges in epitope variability and functional redundancy demand innovative strategies. By integrating evolutionary, structural, and immunological insights, this review provides a roadmap for leveraging PE/PPE biology to develop next-generation TB interventions.

## 1 Introduction

Tuberculosis (TB), caused by *Mycobacterium tuberculosis* (Mtb), remains one of the world’s most persistent infectious diseases, with a disproportionate burden in low-resource settings (1). The World Health Organization (WHO) estimates that, in 2023, there were 10.8 million new TB cases and 1.25 million deaths globally, positioning TB as one of the top ten causes of death worldwide (2). The emergence of multidrug-resistant tuberculosis (MDR-TB) and TB associated with HIV further complicates efforts to control the disease, exacerbating the global public health crisis. Among the various strategies to combat TB, vaccination remains the most cost-effective approach (3). However, despite the use of the Bacille Calmette-Guérin (BCG) vaccine since 1921, no new, effective vaccines have been developed. While BCG provides substantial protection against childhood TB, its efficacy in adults and adolescents remains limited (4). Moreover, the effectiveness of BCG varies significantly across regions, influenced by factors such as climate, economics, and healthcare infrastructure (5). In response, the WHO has launched initiatives aimed at developing the next generation of TB vaccines.

The PE/PPE proteins of mycobacteria—defined by the highly conserved N-terminal Pro-Glu (PE) or Pro-Pro-Glu (PPE) motifs—represents a unique and highly diverse group of proteins (6). In Mtb, the 169 genes encoding PE and PPE proteins constitute approximately 7–10% of the genome’s coding capacity (7). Although similar genes exist in fast-growing mycobacteria, their amplification and functional specialization are particularly pronounced in slow-growing species (8). These proteins are predominantly secreted to the bacterial surface or extracellular space via the Type VII secretion system (T7SS) (9). PE/PPE proteins play essential roles in both bacterial physiology and host-pathogen interactions. In terms of bacterial physiology, they are involved in nutrient uptake, metabolism, enzymatic activity, and drug resistance (10, 11). In host-pathogen interactions, they modulate processes such as phagosome maturation, antigen presentation, autophagy, cell death regulation, and both innate and adaptive immune responses, interacting with Toll-like receptors (TLRs) (11). Importantly, PE/PPE proteins are rich in T-cell and B-cell epitopes, eliciting strong immune responses and thus representing promising candidates for diagnostic tools and subunit vaccine development (12).

Despite the biological significance of the PE/PPE family, considerable challenges remain in their study. The redundancy and sequence repetition inherent in these proteins complicate functional annotation (6). Their high content of low-complexity sequences further complicates structural analyses, limiting our understanding of their three-dimensional conformation and functional domains. Different PE/PPE proteins—or even distinct domains within the same protein—may have divergent roles in modulating host immunity, and the flexible interactions between these proteins contribute to their functional diversity, adding complexity to their study (11, 13). Although recombinant protein expression systems, such as *Mycobacterium smegmatis* (*M. smegmatis*), are commonly used for functional analysis, these models may fail to replicate the native localization and functionality of PE/PPE proteins in Mtb. Additionally, the limitations of animal models—ranging from mice to non-human primates—pose challenges in assessing vaccine efficacy, as these models often reflect only certain immunological features of TB (14, 15). Nevertheless, vaccines targeting PPE proteins, such as M72/AS01E and ID93/GLA-SE, have shown promise in clinical trials, providing valuable insights for the future development of PE/PPE-based vaccines.

Recent advances in evolutionary biology, functional genomics, and structural biology have significantly enhanced our understanding of the PE/PPE family. This review aims to synthesize these developments, focusing on the characteristics, classification, evolution, structure, subcellular localization, secretion mechanisms, and physiological and pathological functions of PE/PPE proteins, as well as their potential as vaccine antigens. By elucidating the diversity and biological relevance of PE/PPE proteins, we aim to lay a theoretical foundation for the next generation of anti-tuberculosis vaccines.

## 2 General characteristics of the PE/PPE protein family

The PE/PPE protein family is a distinctive and multi-domain protein family exclusive to mycobacteria (7). In the Mtb H37Rv reference strain, the genome encodes 100 members of the PE family and 69 members of the PPE family (6). The number of PE/PPE genes can vary across different Mtb clinical isolates, reflecting both genetic polymorphism and strain-specific expression patterns. The genomic distribution of *pe/ppe* genes in Mtb is notably non-random. At least 28 distinct *pe/ppe* operons have been identified, such as *pe25-ppe41* and *pe35-ppe68*, with the majority of classical *pe* genes (excluding *pe_pgrs* members) located upstream of their corresponding *ppe* genes within these operons. By contrast, *pe_pgrs* genes are dispersed throughout the genome and generally do not form operonic structures with *ppe* genes, suggesting functional and regulatory divergence within the *pe/ppe* family (16, 17). Furthermore, these genes are often clustered near the ESX (ESAT-6 secretion system) gene clusters, particularly those associated with the ESX-1 and ESX-5 transcriptional units, suggesting an evolutionary and functional link with these secretion systems (8).

A defining feature of the PE/PPE family is the high GC content of their gene sequences, which results in relatively low transcriptional and translational efficiency. This may serve as a mechanism for tightly regulated gene expression in response to various environmental stresses encountered during infection (6). These stressors include conditions such as hypoxia, nutrient deprivation, heat shock, and acidic pH, all of which influence the tissue-specific expression of these genes during the infection process (18, 19).

The PE/PPE protein family is characterized by significant gene redundancy and polymorphism, particularly in their C-terminal regions. Many *pe/ppe* genes contain variable numbers of tandem repeat sequences (VNTRs), which are subject to mutations through mechanisms like homologous recombination, gene conversion, and repeat expansion. These genetic mechanisms provide the PE/PPE proteins with substantial adaptive potential (20). Among these, the PE_PGRS (Polymorphic GC-Rich Sequence) proteins exhibit the highest degree of polymorphism. A comparative analysis of 27 *pe_pgrs* genes across 94 clinical isolates revealed considerable genetic variation, particularly in comparison to other genomic regions. Nucleotide diversity, along with insertion/deletion events and distinct dN/dS ratios, indicates diverse selective pressures acting on these genes (21). Furthermore, a recent study has shown that *pe/ppe* transcripts are enriched in RNA G-quadruplex (rG4) structures, suggesting an additional layer of post-transcriptional regulation. These rG4 elements may enable Mtb to fine-tune PE/PPE protein expression in response to infection stage and environmental stress, thereby modulating the intensity of host–pathogen interactions (22).

From an immunological perspective, the PE/PPE proteins exhibit varying degrees of sequence conservation and variability, resulting in marked differences in their immunogenic potential. The N-terminal domains of these proteins are typically more conserved and enriched in T-cell epitopes, whereas the C-terminal regions, especially within the PE_PGRS and PPE_MPTR (Major Polymorphic Tandem Repeats) subfamilies, display higher genetic variability (7, 21). Additionally, antibodies against multiple PE/PPE proteins have been detected in the sera of TB patients, suggesting that these proteins are recognized by the immune system and can elicit humoral responses during natural infection (23, 24). Initially, these proteins were thought to primarily contribute to antigenic variation as a mechanism to evade host immune surveillance (25). However, emerging evidence indicates that not all pe/ppe genes are under positive selective pressure; many instead exhibit characteristics of neutral evolution or purifying selection, challenging the traditional view of antigenic variation as the primary driver of their evolution (20, 21).

## 3 Classification of the PE/PPE protein family

The PE/PPE protein family can be classified through several approaches, including analysis of C-terminal diversity, phylogenetic relationships, and functional roles.

A defining characteristic of the PE protein family is the considerable variability observed in the C-terminal region, which serves as a key basis for classification (6, 8). The PE family is divided into two subfamilies: PE and PE_PGRS (Polymorphic GC-Rich Sequence). PE proteins possess a conserved N-terminal structure with a relatively short, less variable C-terminal, whereas PE_PGRS proteins feature extended C-terminals rich in repeat sequences, such as glycine-rich polymers (Gly-Gly-Ala or Gly-Gly-Asn), contributing to their high variability (6, 8). Phylogenetic analysis further subdivides the PE family into five distinct subfamilies. Subfamilies I and II, representing the oldest members, include PE34 and PE35 (subfamily I) and PE5 and PE15 (subfamily II). Subfamily III includes PE22, PE25, and PE36, while subfamily IV contains PE proteins secreted via the ESX-5 system, which are pivotal in Mtb-host cell interactions. Subfamily V is primarily composed of PE_PGRS proteins, with some members containing unique C-terminal enzymes such as lipases (8, 11).

The classification of the PPE family is also determined by the diversity of the C-terminal region, with PPE proteins typically possessing extended C-terminals that vary significantly in structure and function. Based on these variations, the PPE family is categorized into four subfamilies: PPE proteins lacking distinctive C-terminal sequences, PPE proteins with the PxxPxxW motif (PPE_PPW), PPE proteins containing the GxxSVPxxW motif (PPE_SVP), and PPE proteins with major polymorphic tandem repeats (PPE_MPTR) (8). Phylogenetic analysis further divides the PPE family into five subfamilies. Subfamily I include members such as PPE68. Subfamily II is composed of PPE_PPW members, while subfamily III includes PPE36, PPE41, PPE57, PPE58, PPE59, and PPE69. Subfamilies III and IV are particularly involved in immune evasion. Subfamily V consists of PPE_MPTR members, which are typically large, with some secreted PPE proteins exceeding 3,000 amino acids in length (8, 26).

## 4 Evolution of the PE/PPE family proteins

### 4.1 Evolutionary differences of PE/PPE family proteins across species

The evolutionary trajectory of the PE/PPE protein family is indicative of the genome plasticity that has enabled *Mycobacterium* species to adapt to diverse ecological niches and develop pathogenicity. Comparative genomic analyses reveal distinct patterns of expansion within these gene families in mycobacterial species with differing lifestyles.

In free-living, fast-growing, non-pathogenic mycobacteria such as *M. smegmatis*, only two pairs of *pe/ppe* genes are present, which are primarily involved in basic metabolic functions (27). In contrast, pathogenic, slow-growing mycobacteria exhibit substantial expansion of these gene families (**Table 1**). For instance, the genome of *Mycobacterium marinum* contains 281 *pe/ppe* genes, encoding 27 PE, 148 PE_PGRS, and 106 PPE proteins (28). Notably, *Mycobacterium leprae* (*M. leprae*) represents an exceptional case: although it is a slow-growing pathogen, it has undergone significant genomic reduction. With a genome size of approximately 3.2 Mb and containing 1,129 pseudogenes, *M. leprae* harbors only 1,440 protein-coding genes, in stark contrast to other closely related mycobacterial species, which possess more than 4,000 protein-coding genes (29). In terms of *pe/ppe* genes, *M. leprae* retains just 9 complete *pe* genes and 10 complete *ppe* genes (30). This genomic reduction reflects the ecological niche contraction that accompanied the transition of *M. leprae* from a free-living ancestor to an obligate intracellular parasite.

**Table 1:** Numbers of *pe/ppe* genes in the genomes of mycobacterial species.

Species	*ppe*	*pe*	*pe_pgrs*	*ppe_mptr*
*Mycobacteroides chelonae* MCHL-2035	9	5	0	0
*Mycobacteroides abscessus* ATCC 19977	7	3	0	0
*Mycolicibacterium arenosum* CAU 1645	3	2	0	0
*Mycolicibacterium arabiense* JCM 18538	5	5	0	0
*Mycolicibacterium smegmatis* MC2 155	2	2	0	0
*Mycolicibacterium aurum* NCTC10437	2	3	0	0
*Mycolicibacterium phlei* NCTC8156	2	2	0	0
*Mycolicibacterium komossense* DSM 44078	5	3	0	0
*Mycolicibacterium helvum* JCM 30396	4	3	0	0
*Mycolicibacter terrae* NCTC10856	42	12	0	0
*Mycolicibacter engbaekii* ATCC 27353	26	11	0	0
*Mycolicibacillus trivialis* DSM 44153	22	7	0	0
*Mycobacterium xenopi* NCTC10042	33	18	0	0
*Mycobacterium kyorinense* HF1629	37	25	0	0
*Mycobacterium gordonae* DSM 44160	87	80	93	37
*Mycobacterium kansasii* ATCC 12478	117	56	51	36
*Mycobacterium marinum* M	106	27	148	31
*Mycobacterium tuberculosis* H37Rv	69	36	64	21
*Mycobacterium leprae* TN	10	9	0	0
*Mycobacterium simiae* JCM 12377	36	12	0	0
*Mycobacterium alsense* DSM 45230	58	50	26	17
*Mycobacterium avium subsp. avium* DSM 44156	36	9	0	1
*Mycobacterium intracellulare* FDAARGOS_1564	44	10	0	0

Genome sequences and annotation data of *Mycobacterium* species were obtained from the NCBI RefSeq database. The *pe/ppe* gene classification followed three criteria based on original RefSeq annotations (primarily generated by the Prokaryotic Genome Annotation Pipeline, PGAP): 1.genes containing PE/PPE domains recorded in the InterPro database; 2. identification of PE/PPE domains using HMMER suite (v3.4) with hmmscan;3.refseq-annotated PE/PPE -related genes exhibiting characteristic sequence features (*ppe* genes: encoding proteins with PPE motif & WXG motif; *pe* genes: encoding proteins PP/DE motif & YXXXD/E motif). All criteria required domain position quality control (i.e., domains/features must occupy appropriate sequence regions rather than random occurrences). Genes meeting any criterion were classified as *pe/ppe*. *ppe_mptr* and *pe_pgrs* subfamilies were predicted based on phylogenetic tree characteristics of domain sequences.

Notably, the evolutionary expansion of the *pe/ppe* gene family is accompanied by a striking enrichment in RNA G-quadruplex (rG4) structures within their transcripts (22). Although *pe/ppe* genes comprise less than 8% of total transcript length, they account for over 50% of all rG4 motifs identified in Mtb transcripts. Comparative analyses further show that rG4 density exceeds putative quadruplex sequence (PQS) density in the genomes of slow-growing pathogenic mycobacteria (rG4/PQS ratio >1), but not in non-pathogenic species (ratio <1) (22). These findings suggest that during the evolution of pathogenic slow-growers, not only did the *pe/ppe* family expand, but their transcripts also acquired increased rG4 content—potentially enabling fine-tuned post-transcriptional regulation in response to the complex intracellular environment of the host.

### 4.2 Co-evolution of PE/PPE family proteins with the ESX system

The evolution of PE/PPE proteins exhibits a co-evolutionary trajectory with the expansion of *esx* gene clusters. Phylogenetic analyses suggest that the ancestral ESX-4 system initially lacked *pe/ppe* genes. Subsequent gene duplication events gave rise to five distinct ESX loci in Mtb (8). The earliest incorporation of *pe35* (Rv3872) and its partner *ppe68* (Rv3873) into ESX-1—Mtb’s second type VII secretion system—marked the origin of these protein families (31). Subsequent duplications led to the emergence of ESX-3, ESX-2, and ESX-5 (8). Comparative genomics between the fast-growing *M. smegmatis* and the slow-growing Mtb supports this evolutionary trajectory. *M. smegmatis* retains *pe/ppe* pairs associated with ESX-1 and ESX-3 but lacks *pe_pgrs*, *ppe_svp* and *ppe_mptr* subfamilies entirely (8). In contrast, ESX-5, the most recently evolved type VII secretion system, is exclusive to slow-growing mycobacteria and coincides with a significant expansion of PE_PGRS, PPE_SVP and PPE_MPTR subfamilies. This suggests that ESX-5 acquisition was a pivotal event in the evolution of mycobacterial pathogenicity (32).

The diversification of the *pe/ppe* gene family is driven by gene duplication, point mutations, and recombination. Notably, the PE_PGRS and PPE_MPTR subfamilies, characterized by repetitive sequences, undergo frequent recombination, generating extensive protein diversity. Karboul et al. demonstrated that homologous recombination rates within *pe/ppe* loci in clinical mycobacterial isolates significantly exceed those of other genomic regions, suggesting these loci as hotspots for genomic plasticity (33).

The co-evolution of PE/PPE proteins with the ESX system is likely shaped by host-pathogen interactions. Slow-growing pathogenic mycobacteria encounter complex immune pressures necessitating prolonged infection, favoring an expanded PE/PPE repertoire that enhances antigenic variation and immune modulation (27). Conversely, free-living, fast-growing mycobacteria retain a streamlined system, sufficient for their ecological niche.

## 5 Structure of the PE/PPE family proteins

The structural features of PE/PPE proteins are central to their functional roles in mycobacteria. Structural studies employing X-ray crystallography, nuclear magnetic resonance, and bioinformatics have provided insights into their molecular organization. While the conserved N-terminal regions of PE/PPE proteins, particularly those involved in heterodimer formation, have been well characterized, the highly variable C-terminal domains remain structurally elusive.

### 5.1 Basic structure of PE/PPE family proteins

PE proteins are defined by a conserved N-terminal domain (~100 amino acids) that adopts an antiparallel α-helix-turn-α-helix conformation. This region harbors the characteristic PE motif (Pro-Glu) and a YXXXD/E secretion signal within the first α-helix. The C-terminal domain exhibits significant variability, ranging from short extensions to >1400 amino acids. Members of the PE_PGRS subfamily contain glycine-rich repeats (e.g., Gly-Gly-Ala, Gly-Gly-Asn), which may confer structural flexibility (25). Recent computational structure predictions of PE_PGRS proteins using AlphaFold revealed the PGRS domain as tightly packed β-sandwiches, prompting the authors to propose a ‘sailing’ model wherein this domain acts as a mechanistic framework to diffuse along the mycomembrane, expose structural motifs mediating host interactions, and deliver functional C-terminal protein modules (34).

PPE proteins share a conserved N-terminal domain (~180 amino acids) composed of five α-helices arranged in a helical bundle. The PPE motif is localized in the first α-helix, while the WxG motif resides between the second and third helices. A hydrophobic hh motif, positioned between the fourth and fifth α-helices, is crucial for interactions with secretion chaperones (35). The PPE C-terminal domain is highly variable, exceeding 3000 amino acids in some cases. Members of the PPE_MPTR subfamily are distinguished by a conserved Asn-X-Gly-X-Gly-Asn-X-Gly (NXGXGXN) repeat motif (6).

### 5.2 PE-PPE heterodimer structure

Although definitive experimental evidence remains limited, PE and PPE proteins are thought to be secreted as heterodimers via the mycobacterial ESX system. Structural elucidation of the PE25/PPE41 heterodimer revealed that the α-helices of PE25 interact with those of PPE41 via hydrophobic and intermolecular forces, forming a stable four-helix bundle (36). This organization juxtaposes the PE YXXXD/E motif with the PPE WxG motif, likely generating a composite secretion signal for type VII secretion system (T7SS) recognition (37). Several PE-PPE pairs, including PE35-PPE68, PE18-PPE26, and PE5-PPE4, have been experimentally validated (16, 31, 38). While most PE-PPE pairs are encoded by adjacent genes, non-adjacent interactions, such as PE19-PPE51, indicate a degree of pairing flexibility (10). Moreover, PE-PE interactions, exemplified by PE9-PE10 surface-localized dimers, further diversify the functional landscape of this protein family (39).

Strikingly, PE-PPE heterodimers share structural homology with ESX substrates, including the EsxA/EsxB (ESAT-6/CFP-10) complex and EspB, folding into multi-helical bundles with conserved secretion-associated motifs. This structural conservation supports their role as canonical T7SS substrates and suggests shared functional mechanisms (40, 41).

### 5.3 PE-PPE-EspG heterotrimer structure

The secretion-associated protein EspG plays a pivotal role in PE-PPE protein export. Structural analysis of the PE25-PPE41-EspG5 complex revealed that EspG5 binds to the PPE hh motif, preventing aggregation and stabilizing the heterodimer during secretion (42). Subsequent studies on PE8-PPE15-EspG5 suggested a conserved interaction interface across PPE proteins (43). Comparative structural analyses indicate that while PE-PPE-EspG complexes share a conserved binding mode, subtle interaction differences exist. For example, in the ESX-3 system, EspG3 binds PPE4 at a distinct angle relative to EspG5 interactions with PPE15 and PPE41. Additionally, PPE4 exhibits an extended hh motif loop, highlighting potential ESX system-specific substrate adaptations (31).

## 6 Subcellular localization and secretion of PE/PPE proteins

The spatial organization and transport of PE-PPE proteins are integral to their functional specialization in mycobacteria. These proteins exhibit distinct subcellular distribution patterns, dictated by specialized secretion systems and molecular chaperones.

### 6.1 Subcellular distribution of PE/PPE proteins

High-throughput proteomic analyses have identified over 35 PE/PPE proteins in the membrane and/or cell wall of Mtb (7). For instance, PE_PGRS33 localizes to the cell wall of *M. smegmatis* and Mtb, guided by its N-terminal PE domain (44–46). Similarly, LipY and PE_PGRS30 utilize N-terminal sequences for secretion and membrane association (47, 48). Notably, PE19-PPE51, PE20-PPE31, and PE15-PPE20 form outer membrane-associated channels, suggesting roles in nutrient exchange or virulence (10, 49–51). Certain PE/PPE proteins are also secreted into the extracellular environment, as evidenced by proteomic detection of at least seven PE/PPE proteins in culture filtrates (7). Recently, Lepe et al. further expanded our understanding of the Mtb surface PE/PPE proteome through the development of protease shaving techniques coupled with quantitative mass spectrometry analysis (52). This study identified 167 proteins with significantly elevated abundance under protease treatment conditions, including multiple PE/PPE family members such as PE12, PE23, PPE10, PPE18, PPE20, PPE32, PPE33, PPE38, PPE40, PPE51, and PPE60. Notably, the researchers validated the surface localization of PPE18, PPE38, and PE23 through flow cytometry, with PPE18, a component of the M72 vaccine candidate, demonstrating particularly remarkable surface enrichment (52).

Intriguingly, subcellular localization may be dynamically regulated by environmental cues. For example, PPE37 undergoes proteolytic cleavage under iron-limiting conditions, leading to differential localization and functional specialization of its N- and C-terminal fragments (13). Such adaptive relocalization may contribute to mycobacterial persistence and host adaptation.

### 6.2 Mechanisms of PE/PPE translocation across the membrane

#### 6.2.1 Inner membrane transport

The translocation of PE/PPE proteins across the inner membrane is orchestrated by the ESX secretion system, a multi-component apparatus that ensures substrate specificity and transport fidelity. PE-PPE heterodimers first associate with EspG, a dedicated chaperone that stabilizes the complex and prevents aggregation via interactions with the PPE hh motif (53, 54).

The ESX membrane translocon comprises five core components—EccB, EccC, EccD, EccE, and MycP—along with cytosolic factors EccA and EspG, forming a ~2 MDa secretion complex (55–57). Cryo-electron microscopy studies of ESX-3 and ESX-5 have provided structural insights into this machinery (56, 58). EccC, the key ATPase, drives substrate translocation via ATP hydrolysis, with its third nucleotide-binding domain (NBD3) interacting with the YXXXD/E secretion signal, while linker 2 mediates PE-PPE specificity (59, 60). Mutational analyses highlight the functional importance of EccC, as disruptions in its NBD1 domain abrogate PE_PGRS secretion (61). EccD, the central transmembrane conduit, features 11 transmembrane helices, while EccB, EccE, and EccC contribute to structural integrity and motor function. The protease MycP stabilizes the complex via a single transmembrane domain (56). Notably, while some PE_PGRS proteins (e.g., PE5, PE15, PE_PGRS12, PE_PGRS29) possess putative Sec-signal sequences, their dependence on the Sec pathway remains unconfirmed (62).

#### 6.2.2 Outer membrane translocation

The mechanisms facilitating PE/PPE protein translocation across the outer membrane remain incompletely understood. Some evidence suggests that PE/PPE proteins contribute to outer membrane channel formation, potentially mediating their own export. In *M. marinum*, PPE68 and its functional homolog MMAR_2894 are essential for ESX-1 substrate secretion, suggesting a role in periplasmic or outer membrane transport (63, 64). However, PPE68 does not appear to form a stable membrane channel but is instead stored intracellularly and subsequently processed by PecABC and other proteases (64).

Recent work expressing the *Mycobacterium xenopi* esx-5 operon in *M. smegmatis* demonstrated that PPE proteins within the esx-5 gene cluster are indispensable for functional ESX-5 secretion, implicating these proteins in outer membrane translocation (32). In Mtb, PPE38 is critical for ESX-5-dependent PE_PGRS and PPE_MPTR secretion; loss-of-function mutations result in secretion defects and altered virulence (65). Strikingly, clinical isolates of the hypervirulent Beijing lineage exhibit *ppe38* deletions, with reintroduction of PPE38 attenuating virulence, further underscoring its functional significance (65, 66). While the precise mechanism remains unresolved, PPE38 may function as a secretion facilitator, either directly forming an outer membrane channel or interacting with auxiliary factors. The ESX-5 system may employ additional, yet unidentified, components to mediate PE/PPE export, warranting further investigation.

## 7 Functions of the PE/PPE family proteins

### 7.1 Biological functions in bacterial physiology

#### 7.1.1 Nutrient acquisition

The highly hydrophobic cell wall of Mycobacterium tuberculosis lacks classical porins for transmembrane transport (11). Recent findings suggest that PE/PPE family proteins function as outer membrane channels mediating nutrient uptake, despite the absence of direct structural evidence. PPE51 and PE19 facilitate the uptake of glucose, glycerol, maltose, trehalose, and certain low-molecular-weight drugs (10, 49, 50). The PE20-PPE31 and PE15-PPE20 complexes mediate magnesium and calcium uptake, respectively (10, 51). The PE5-PPE4 complex, a substrate of the ESX-3 secretion system, is crucial for mycobactin-bound iron uptake; its deletion renders Mtb incapable of growth under low-iron conditions (67). This system is transcriptionally regulated by the metal-ion-responsive transcription factors Zur and MntR (68, 69). However, no evidence currently supports the involvement of PE5-PPE4 in Zn or Mn uptake.

While Mitra et al. identified PPE36, along with PPE62, as essential for heme utilization (70, 71), Tullius et al. reported no impairment in heme uptake following *ppe36* deletion (72). Instead, their findings implicated PPE37 in heme acquisition (72). This discrepancy may arise from strain differences, as Mitra et al. used H37Rv, whereas Tullius et al. employed the Erdman strain. Given the variability of *ppe37* among clinical isolates (72, 73), PPE37 is unlikely essential for Mtb survival. Additionally, PPE37 and its paralogs contain a positively charged C-terminal segment that may serve as a nuclear localization signal (NLS), suggesting potential novel functions (13). PPE64 also exhibits heme-binding ability and channel-forming activity, likely contributing to heme-iron utilization (74). The cell wall-localized PE-PGRS3, with an arginine-rich C-terminal, interacts with negatively charged phospholipids on alveolar epithelial cells, facilitating phosphate acquisition under nutrient-limiting conditions (75).

#### 7.1.2 Metabolism and enzyme activity

During infection, Mtb enters dormancy within alveolar macrophages, reactivating upon immune suppression. Dormancy maintenance and reactivation depend on the storage and metabolism of fatty acids and cholesterol within nutrient-limited phagolysosomes (76). PPE15 (mper1), upregulated during dormancy, is crucial for triglyceride accumulation and homeostasis (77). Deletion of *mper1* prevents lipid droplet formation, thereby impairing dormancy establishment both *in vitro* and in human granuloma models (78). PE_PGRS63 (LipY), a member of the hormone-sensitive lipase family, preferentially hydrolyzes short to intermediate p-nitrophenyl esters and is highly induced under starvation and hypoxia. It serves as the primary lipase for stored triglyceride utilization, playing a critical role in dormancy exit (79, 80).

The surface-localized esterase PE11 (LipX) influences cell wall remodeling and virulence; its deletion alters cell wall composition and reduces intracellular survival within macrophages (81). In *M. smegmatis*, recombinant PE11 modulates fatty acid profiles in cell wall polar lipids, increasing hydrophobicity and enhancing resistance to stressors such as SDS, lysozyme, acidity, and anti-tuberculosis drugs (24). Other PE proteins, including PE16, PE1, and PE2, exhibit serine esterase activity *in vitro*, though their role in virulence remains unclear (82, 83). The C-terminal domain of PPE63 also possesses esterase activity, potentially modulating cell wall properties by altering lipid composition (84).

### 7.2 Functions in regulating host immune responses

#### 7.2.1 Interaction with TLRs to modulate downstream signaling

**Figure 1:**
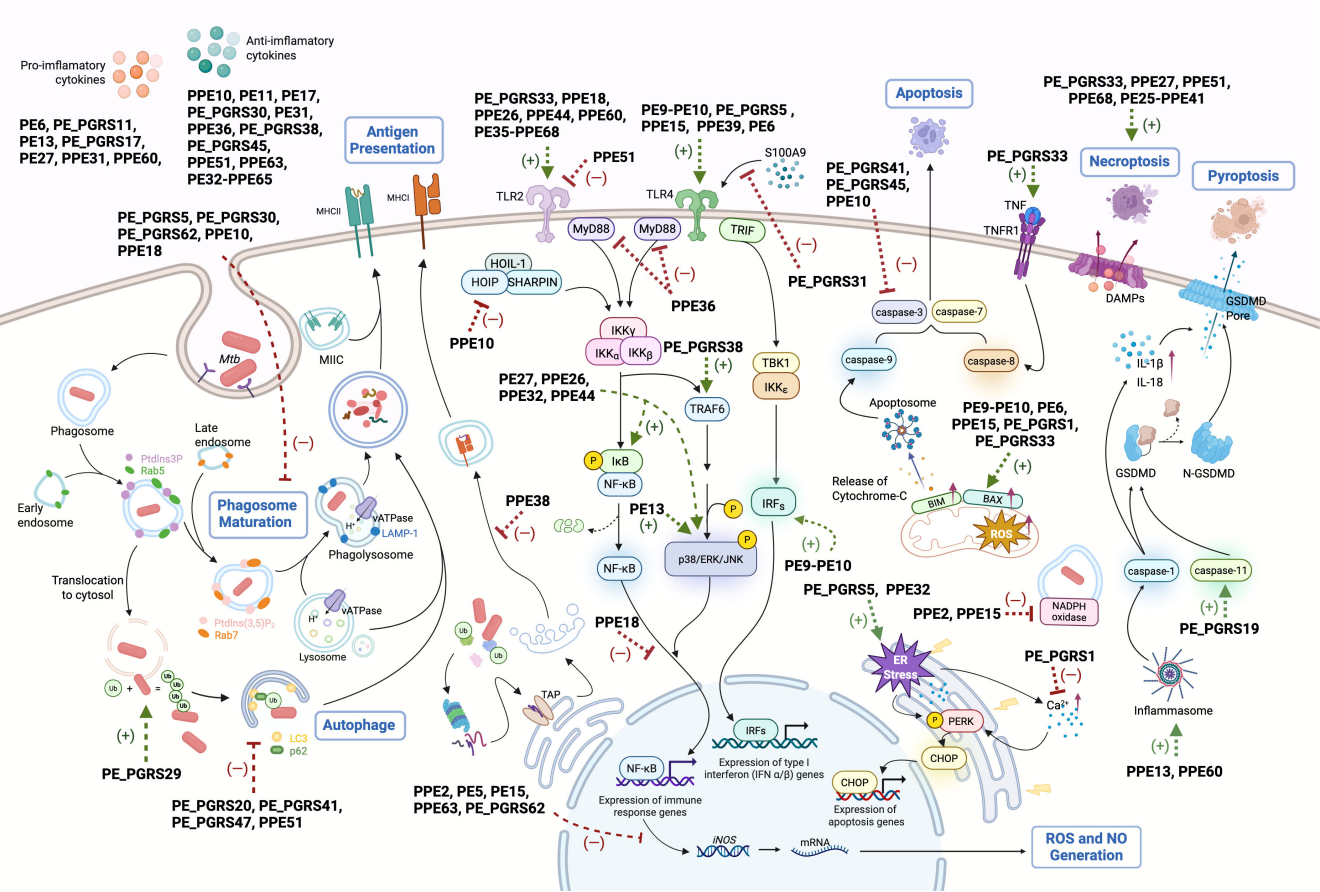
Schematic representation of PE/PPE-mediated immunomodulatory mechanisms in host immune cells. PE/PPE family proteins mediate diverse immunomodulatory functions across multiple phases of Mtb-host interactions. Key mechanisms include: (1) Toll-like receptor (TLR) engagement to modulate downstream signaling cascades; (2) Disruption of phagosome maturation; (3) Interference with antigen presentation; (4) Neutralization of macrophage-derived reactive oxygen species (ROS)/nitric oxide (NO); (5) Regulation of autophagy through ubiquitin (Ub)-dependent pathways; (6) Modulation of programmed cell death; (7) Cytokine network manipulation; (8) Shaping of adaptive immunity. Green arrows: PE/PPE-induced processes, red blunt lines: PE/PPE-inhibited pathways.

During early infection, host pattern recognition receptors, particularly Toll-like receptors (TLRs), recognize mycobacterial ligands, initiating immune responses. Several PE/PPE proteins interact with TLRs, notably TLR2 and TLR4, to influence immune signaling cascades (**Figure 1**).

PE_PGRS33 promotes Mtb entry into macrophages via a TLR2-dependent mechanism, activating the PI3K-mediated adhesion pathway (85). Inactivation of PE_PGRS33 in BCG Pasteur results in impaired bacterial growth in liquid medium and macrophages (86). Similarly, PPE26 and PPE44 activate TLR2-mediated p38 MAPK and NF-κB signaling, inducing IL-6 and IL-12p40 production in a dose-dependent manner (87, 88). PPE60 promotes dendritic cell maturation and Th1/Th17 responses via TLR2 activation (89). In contrast, PPE51 acts as a TLR2 antagonist, inhibiting autophagy, cytokine secretion, and antigen presentation (90). Mtb also exploits TLR2 signaling to suppress host immunity; PPE18 triggers TLR2-mediated p38 MAPK activation, enhancing IL-10 secretion and attenuating bacterial virulence (91, 92). The PE35-PPE68 complex similarly induces IL-10 and MCP-1 while suppressing IL-12p40 via TLR2-mediated MAPK activation (93). PPE36 inhibits NF-κB activation through Smurf1-mediated MyD88 degradation, although direct PPE36-TLR interactions remain unconfirmed (94).

Several PE/PPE proteins modulate TLR4 signaling. The PE9-PE10 complex activates the TRIF pathway via TLR4, enhancing IFN-β secretion (39). PE_PGRS31 inhibits TLR4-MyD88-NF-κB signaling by blocking S100A9 binding, promoting Mtb survival (95). Conversely, PE6 and PPE15 interact with TLR4 to upregulate NF-κB and induce TNF-α and IL-1β secretion (96, 97). PPE39 enhances DC maturation and Th1 polarization via TLR4 (98).

#### 7.2.2 Interference with phagosome maturation

Host macrophages are the preferred niche of Mtb. Once macrophages internalize pathogens, the phagocytic vesicles need to fuse with lysosomes and mature to create an acidic environment that kills pathogens (99). Mtb can successfully avoid phagosome maturation by manipulating multiple strategies, with some PE/PPE involved. PE_PGRS30 inhibits phagosome-lysosome fusion by reducing LAMP-1 expression, and its deletion lowers bacterial burden and attenuates lung pathology in mice (47, 100). PE_PGRS5 and PE_PGRS62 promote survival under acidic stress by suppressing Rab7 and cathepsin D (101, 102). Homologs of these proteins in *M. marinum* are enriched in granulomas, suggesting conserved roles in persistence (103). While *M. avium* MAV_2928 blocks phagosome-lysosome fusion, functional evidence for Mtb’s PPE25 remains elusive (104, 105).

#### 7.2.3 Interfering with antigen presentation

Mtb with PE_PGRS47 mutation exhibits enhanced MHC II-restricted antigen presentation during infection in mice. Intriguingly, of the tested antigens, PE_PGRS47 was found to inhibit the presentation of TB9.8 and Ag85B P25, but not ESAT-6, indicating that PE_PGRS47 was ineffective during the early stages of infection when ESAT-6 was expressed, or because of that ESAT-6 can directly break down the phagosome membrane and access other cellular compartments (106). Studies involving the *M. marinum* PPE38 knockout strain and PPE38 recombinant *M. smegmatis* indicated that PPE38 decreases the expression of MHC class I proteins, and consequently, the count of effector/memory CD8 T-cells in mice (107–110). Given that PPE38 is among the most highly expressed proteins in Mtb after 90 days of infection in guinea pigs, it may play a significant role in infection persistency (80).

#### 7.2.4 Counteracting macrophage ROS and NO killing

PE-PPE proteins neutralize ROS/NO defenses. PPE2 translocates to host nuclei via an NLS, repressing iNOS transcription and sequestering p67phox to inhibit NADPH oxidase assembly (111, 112). MtbΔPPE2 exhibits reduced virulence in mice (112). PE5, PE15, PPE63 and PE_PGRS62 similarly suppress iNOS/ROS in *M. smegmatis* and *M. marinum* (102, 113). PE13 enhance bacterial survival under oxidative stress, correlating with stress-induced expression (114).

#### 7.2.5 Regulating cell death

##### 7.2.5.1 Apoptosis

While macrophage apoptosis restricts Mtb growth and promotes antigen cross-presentation, the pathogen exploits apoptosis for dissemination via apoptotic bodies (115). PE/PPE proteins regulate apoptosis through ER stress, mitochondrial pathways, and TNF signaling. PE_PGRS5 induces caspase-8-dependent apoptosis via ER stress, whereas PE_PGRS62 suppresses it (116, 117). PE9-PE10 and PE_PGRS33 activate mitochondrial pathways (e.g., Bax upregulation, caspase-3) or TNFRI signaling (39, 118, 119). Conversely, PE_PGRS1 inhibits apoptosis by blocking PERK-mediated stress (120).

##### 7.2.5.2 Necrosis

Necrosis facilitates bacterial dissemination and is exploited by Mycobacterium tuberculosis (Mtb) via PE-PPE proteins. Mtb Δ *ppe51* strain induces necrosis via elevated ROS, exceeding levels in wild-type or complemented strains (90) PE_PGRS33 amplifies necrosis in *M. smegmatis*-infected macrophages by elevating TNF, reflected in increased LDH and nucleosome release (121). The PE25-PPE41 complex drives dose-dependent necrosis independently of TNF-α, NO, or NF-κB signaling (122). PPE68 and PPE27 promote necrosis, as Mtb Δ*ppe68* reduces LDH release, while Msmeg-PPE27 elevates it (123, 124) However, LDH and Annexin V/PI staining alone cannot exclude concurrent apoptosis or pyroptosis.

##### 7.2.5.3 Pyroptosis

PE/PPE proteins drive pyroptosis to amplify inflammation. PPE13 activates NLRP3 inflammasome-mediated pyroptosis, while PE_PGRS19 enhances caspase-11-dependent GSDMD cleavage (125, 126). PPE60 upregulates NLRP3 and GSDMD, suggesting a pro-pyroptotic role (127).

##### 7.2.5.4 Autophagy

PE/PPE proteins modulate autophagy to balance bacterial persistence and host defense. PPE51 inhibits autophagy; its deletion enhances autophagic flux and reduces Mtb burden (90). PE_PGRS47 and PE_PGRS20 suppress autophagy by disrupting Rab1A-ULK1 interactions, impairing autophagosome formation (106, 128). PE6 inactivates mTORC1-ULK1 signaling, while PE_PGRS41 blocks LC3-II conversion (96, 129). Paradoxically, the surface-localized PE_PGRS29 promotes host autophagy by recruiting ubiquitin and engaging ubiquitin-binding receptors such as p62 and NBR1, potentially modulating intracellular bacterial burden to facilitate persistent infection (130).

#### 7.2.6 Modulation of cytokine production

##### 7.2.6.1 Cytokine dynamics in infection

Early Mtb infection triggers IL-1β production by macrophages and dendritic cells (DCs), essential for granuloma formation through macrophage migration to lymph nodes (131). While critical for initiating adaptive immunity, excessive IL-1β exacerbates tissue damage during chronic phases (132). TNF-α maintains granuloma integrity by promoting lymphocyte-macrophage clustering and enhancing NADPH oxidase-mediated ROS production (109, 133). IL-6 exhibits dual roles: early deficiency increases susceptibility, yet sustained expression impairs Th1 polarization and IFNγ production (110, 134, 135).

Th1 responses dominate chronic-phase protection, though Th17 contributions emerge through IL-17-mediated neutrophil recruitment and CXCL13-driven lymphoid organization (136, 137). While IL-12p40 from APCs drives Th1 polarization, IL-10 suppresses this process by blocking IL-12p40 production (138). Late-stage IL-10R1 blockade enhances bacterial control in mice, suggesting temporal regulation of IL-10’s immunosuppressive effects (139–141). Paradoxically, IL-23/Th17 axis amplifies IL-12p40 secretion but risks pathological inflammation through neutrophil infiltration (142, 143).

##### 7.2.6.2 Proinflammatory cytokine induction

Mtb virulence factors orchestrate proinflammatory responses through distinct signaling mechanisms. PE13 enhances IL-6/IL-1β production in macrophages via p38/ERK/NF-κB activation while suppressing SOCS3 expression (114). PPE60 promotes Th1/Th17 polarization through DC-derived IL-12p70 and IL-23p19 (89, 127), whereas PE6 modulates TLR4 signaling to elevate TNF-α/IL-6/IL-12 levels (96, 144). Structural PE_PGRS proteins (Rv0978c, Rv0754) induce DC maturation and proinflammatory cytokine secretion, correlating with CD4+ T-cell activation (145). PE27 specifically activates MAPK/NF-κB pathways to drive TNF-α/IL-6/IL-1β production (146). These coordinated mechanisms suggest evolutionary optimization of Mtb’s capacity to manipulate host inflammatory cascades.

##### 7.2.6.3 Anti-inflammatory modulation

Mtb counterbalances inflammation through sophisticated immunosuppressive strategies. PPE51 deletion elevates IL-6/IL-1β/ROS and impairs bacterial survival, revealing its anti-inflammatory function (90). PE_PGRS38 destabilizes TRAF6 via HAUSP-mediated interference with K48-polyUb deubiquitination, suppressing TNF-α/IL-6/IL-1β to enhance intracellular persistence (147). PPE10 inhibits NF-κB by downregulating LUBAC component HOIP (148), while PE_PGRS45/PE31/PPE36 shift cytokine profiles toward IL-10 dominance (149–151). PE11 exacerbates tissue damage through TNF-α/Th2 cytokine induction (24), contrasting with PPE65/PE32-PPE65’s dose-dependent IL-10 promotion and IL-6 suppression (152). This multi-layered regulation enables Mtb to establish chronic infection by modulating both pro- and anti-inflammatory axes.

### 7.3 Roles in drug resistance

Emerging evidence suggests that PE/PPE family proteins may contribute to drug resistance in Mtb. Early whole-genome analyses of drug-resistant clinical isolates identified mutations in *pe/ppe* genes in the absence of canonical resistance-conferring mutations. For example, certain kanamycin-resistant strains lacking alterations in *rrs*, *rpsL* or *eis* were found to harbor mutations in *ppe60*. Similarly, mutations in *pe_pgrs9* or *ppe54/55* were observed in pyrazinamide-resistant strains without known resistance mutations (153). Subsequent analysis of 161 drug-resistant Mtb isolates revealed that several *pe/ppe* genes—including *pe_pgrs4*, *pe_pgrs9*, *ppe13*, *ppe20*, and *ppe9*—exhibited significantly elevated mutation frequencies in resistant compared to susceptible strains (154). Enrichment of mutations was also reported in *pe_pgrs* genes (*pe_pgrs*3, 6, 9, 10, 19, 33, and 49) among 37 extensively drug-resistant (XDR) strains from Pakistan (155). In parallel, variants in *ppe18*, *ppe19*, *ppe46*, and *ppe47* were found to be associated with the spread of isoniazid resistance (156).

Gene interaction studies further implicated PE/PPE proteins in resistance phenotypes. Resistance-associated gene pairs frequently included a known drug target and a *pe/ppe* gene, such as *katG*–*ppe54* and *rpoB*–*ppe54* (isoniazid and rifampicin resistance, respectively), and *embA*–*ppe68* and *embB–ppe54* (ethambutol resistance) (157). A large-scale analysis of 1,170 clinical isolates found that 36% of homoplastic SNPs—variants recurrent across independent lineages—resided in *pe/ppe* genes, and identified a novel mutation in *pe_pgrs*7 linked to streptomycin resistance (158).

While the mechanistic basis of these associations remains unclear, these findings suggest that *pe/ppe* mutations may modulate drug susceptibility. Functional studies support this hypothesis: heterologous expression of PE/PPE proteins, such as PPE63 (84), PE11 (24), and PE_PGRS41 (129), in *M. smegmatis* altered cell wall lipid profiles and surface hydrophobicity, impacting drug permeability. However, whether such changes directly contribute to resistance in Mtb warrants further investigation.

### 7.4 Considerations in studying the functions of PE/PPE proteins

Although numerous studies have reported diverse roles for PE/PPE proteins in mycobacterial physiology and host interactions, several limitations warrant caution in interpreting these findings. Many investigations rely on overexpression or heterologous expression of PE/PPE proteins in *M. smegmatis*, a species that lacks the ESX-5 secretion system required for the proper export of most native PE/PPE proteins (159). These strategies can produce nonspecific effects due to protein aggregation or cellular stress, potentially leading to artefactual phenotypes and misleading suggestions. Subcellular localization remains unresolved for many PE/PPE proteins, further complicating functional interpretation (160). Engineered *M. smegmatis* with functional ESX-5 may help bridge this gap (32).

In addition, many investigations remain descriptive, offering limited mechanistic insight and often failing to reconcile conflicting findings. Several PE/PPE proteins are critical for maintaining mycobacterial cell wall integrity, particularly those with intrinsic enzymatic activity—including lipases and glyco- or proteohydrolases—raising the possibility that phenotypes observed in gene deletion mutants reflect indirect alterations in the bacterial surface, rather than direct modulation of host immunity. *Ex vivo* models—including the treatment of host cells with purified proteins or the ectopic expression of bacterial genes in host systems—often ignore the native expression levels, spatial distribution, and secretion dynamics of PE/PPE proteins during infection. These limitations can obscure the physiological relevance of observed effects and overlook the essential role of bacterial secretion machinery in delivering these proteins.

Functional redundancy across PE/PPE family members also complicates interpretation of single-gene knockout studies, although such models remain essential for delineating context-specific functions within the broader protein network. Recent evidence emphasizes the biological relevance of co-regulated PE–PPE operons—such as PE35–PPE68 and PE25–PPE41—whose co-expression enhances solubility and amplifies immune responses relative to individual proteins (122, 161). This partnership flexibility may enable dynamic host adaptation through stage-specific interactions (11).

Future work should prioritize systems-level approaches to uncover coordinated functions, temporal regulation, and functional cooperativity among PE/PPE proteins. Accurate modeling of native expression and secretion will be critical to advancing our understanding of their multifaceted roles in Mycobacterium tuberculosis pathogenesis.

This review synthesizes current knowledge of PE/PPE functions based on published literature and adopts a functional classification scheme for clarity. However, this framework is necessarily artificial and may not fully capture the biological diversity or contextual complexity of these proteins.

## 8 Vaccine potential of the PE/PPE family proteins

The failure of some individuals to control Mtb infection highlights the need for improved vaccines (3). PE/PPE-based subunit vaccines combine immunodominant T-cell epitopes from selected PE/PPE proteins with other antigens, delivered via adjuvants or vectors (162). These proteins are compelling vaccine targets due to their dense T-cell epitopes and capacity to induce cross-reactive immunity. PPE proteins, in particular, contain numerous confirmed and predicted MHC-binding epitopes that drive robust T-cell responses in humans and animal models, serving as key T-cell targets during Mtb infection (11, 159, 163). Furthermore, epitope redundancy among PE/PPE proteins enables broad CD4+ T-cell cross-reactivity, potentially sustaining immune responses despite shifting PE/PPE expression during infection (164, 165). Current progress in PE/PPE-based vaccine development—spanning protective and therapeutic candidates—is summarized below (**Table 2**).

**Table 2:** Immunoprotective effects of PE/PPE proteins and their associated TB vaccines.

Name of vaccine	Type	Basis	Stage	Features
M72/AS01E	Subunit Vaccine	PPE18, PepA	Phase IIb clinical trials (166)	It offers a 49.7% protection rate in phase IIb trials, but the effectiveness varies across regions (166); In preclinical stage, it shows protective effects in C57BL/6 mice (167), guinea pigs (167, 168), rabbits (169) and cynomolgus macaques (170), where it elicits both Th1 and Th17 responses (171).
ACP	Subunit Vaccine	Ag85B, CFP21, PPE18	Laboratory Research (172)	It significantly induces IFN-γ+ T lymphocytes and IFN-γ secretion. it shows some protective effects in mice, but fails to enhance BCG (172).
ID93/GLA-SE	Subunit Vaccine	Rv1813-Rv3620-Rv3629-PPE42	Phase IIa clinical trials (173)	It elicits strong Th1 and relatively weak Th17 responses in C57BL/6 mice, guinea pigs, and cynomolgus macaques (174, 175). It shows significant efficacy in animal models when administrated as BCG-booster vaccine (174, 176) or therapeutic vaccine (177).
Ag85B-EAST-6-PPE42-rBCG	Recombinant BCG	Ag85B-EAST-6-PPE42	Laboratory Research (178)	It enhances the Th1 while reducing Th2 responses (178).
TriFu64	Subunit Vaccine	PPE42-Rv1793-Rv2628	Laboratory Research (179)	It brings slight alleviation in the lungs of infected mice, but also causes noticeable weight loss (179).
rPPE44	Subunit Vaccine	PPE44	Laboratory Research (180)	It promotes CD4+ T-cell proliferation and IFN-γ secretion, and significantly reduces the bacterial load in the lungs of mice (180), but may have limitations as single-protein-based vaccine.
HPE	Subunit Vaccine	HspX-PPE44-EsxV	Laboratory Research (181–183)	It induces strong secretion of multiple cytokines including IFN-γ in mice when administrated alone or after BCG (181).When combined with resiquimod adjuvant to create HPERC, the cytokine release is furthered enhanced (183).
A3	Subunit Vaccine	Ag85B-PPE57	Laboratory Research (184–186)	It excels in stimulating Th1-type immunity and antibodies production (185), demonstrates protective effects in mice (184), and performs well as a BCG-booster vaccine in mice (186).
A39	Subunit Vaccine	Ag85B-PPE57-Rv2029c	Laboratory Research (187)	It induces protective immunity in pre-exposure mice, but also demonstrates efficacy in the post-exposure model (187).
Tetrafu56	Subunit Vaccine	EspC-TB10.4-PPE57-Hsp-X	Laboratory Research (188)	It induces high levels of IFN-γ from PBMCs of active pulmonary TB patients. It fails to exhibit protective effects, but may serve as a therapeutic vaccine (188).
PPE68-rBCG	Recombinant BCG	PPE68	Laboratory Research (189)	It induces a higher Th1 response without increasing the virulence of BCG (189).
rLmMtb9Ag	Listeria vector	Nine antigens including PPE68	Laboratory Research (190)	It stimulate the proliferation of CD4+ and T-cells in *Mtb* aerosol-challenged C57BL/6 mice, BALB/c mice, and guinea pigs, and reduces the bacterial load. It elicits protective immunity in guinea, pigs but the efficacy does not surpass that of BCG (190).
ChAdOx1.PPE15	chimpanzee adenovirus vector	PPE15	Laboratory Research (191)	It enhances the protective efficacy of BCG in C57BL/6 mice by promoting the CD4+ and CD8+ T-cells’ proliferation (191).
MTB41	Subunit Vaccine	PPE14	Laboratory Research (192)	It demonstrates comparable immunoprotective effects to BCG in both mouse and guinea pig animal models (192).

### 8.1 PPE18

The M72/AS01E subunit vaccine (GlaxoSmithKline), containing a fusion protein (M72, derived from PPE18 and PepA) and AS01E adjuvant, represents a leading post-BCG tuberculosis vaccine candidate. A phase IIb trial involving 3,573 HIV-negative adults in South Africa, Zambia, and Kenya demonstrated 49.7% efficacy over three years in preventing latent-to-active TB progression, though efficacy varied regionally (166, 193). Currently in phase III trials, M72 faces challenges due to PPE18 variability across clinical strains, particularly in putative T-cell epitope regions (194, 195). Computational analyses, however, reveal conserved T-cell epitopes under positive selection, contrasting with hypervariable B-cell epitopes—a disparity that may compromise clinical recognition by M72-induced immunity (73, 196, 197).

Despite incomplete understanding of TB-protective immunity and inconsistent vaccine evaluation frameworks (198), M72 preclinical data offer critical insights. The vaccine conferred protection in murine, guinea pig, rabbit, and cynomolgus macaque models (167–170). In guinea pigs, M72 alone matched BCG’s one-year efficacy, while BCG coadministration extended survival beyond two years post-challenge (168). Macaque studies linked protection to elevated Th1 cytokines (IFN-γ, TNF-α, IL-2), suppressed Th2 signals (IL-4, IL-5), and elevated IFN-γ/IL-6 ratios (170). Phase I trials showed M72/AS01E drives durable M72-specific CD4+ T-cells co-expressing IFN-γ, IL-2, TNF-α, and/or IL-17 (171). Its efficacy may stem from balanced Th1/Th17 induction—critical for chronic infection control (199).

An alternative peptide vaccine (ACP: Ag85B_12-26_, CFP21_12-26_, PPE18_149-163_ epitopes) boosted IFN-γ+ T-cells and antibodies in mice but provided limited lung CFU reduction and failed to synergize with BCG (172), underscoring the need for biomarkers beyond IFN-γ.

### 8.2 PPE42

PPE42 (Rv2608) is a promising vaccine target. The ID93/GLA-SE subunit vaccine combines four Mtb antigens (PPE42, Rv3619, Rv3620, Rv1813) with the TLR4 agonist GLA-SE. Preclinically, it drives Th1 responses and modest IL-17 production in mice, guinea pigs, and macaques, while enhancing BCG’s long-term efficacy. In BCG-primed guinea pigs, ID93/GLA-SE prevented lung lesions for 432 days post-challenge, matching M72/AS02A (168, 174). ID93/GLA-SE-boosted/BCG-primed mice exhibited sustained Th1 CD4+ T-cell and IgG responses, reducing lung/spleen bacterial loads and inflammation after Mtb challenge (176).

Phase I trials in the U.S. and South Africa confirmed ID93/GLA-SE safety and robust Th1/IgG induction in BCG-naive and -vaccinated adults (200, 201). PPE42 dominated T-cell responses, while Rv1813 elicited stronger IgG (201). A Phase 2a trial highlighted its potential to improve TB treatment outcomes (173). As a chemotherapy adjunct, ID93/GLA-SE increased survival in mice and reduced bacterial loads/lesions in macaques (177).

The recombinant 85B-ESAT-6-PPE42-rBCG vaccine elevated Th1 cytokines and T-cell proliferation in mice but lacks *in vivo* protection data (178). TriFu64, a tri-antigen fusion (EsxN, PPE42, Rv2628), modestly lowered murine lung bacterial loads but induced weight loss, potentially linked to elevated TNFα/IL-17 ratios (179).

### 8.3 PPE44

PPE44 (Rv2770c) drives macrophage secretion of proinflammatory cytokines (IL-12 p40, IL-6) and Th1 responses, linked to its high expression across Mtb infection stages and utility as a cross-protective epitope source (88, 180). The gene is conserved clinically, with elevated expression in Beijing genotype strains versus H37Rv (202).

A recombinant PPE44 (rPPE44) subunit vaccine with DDA adjuvant matches BCG’s protection in mice, reducing lung bacterial loads via CD4+ T-cell proliferation and IFN-γ production (180). To overcome single-antigen limitations, multi-fusion strategies emerged. The HspX-PPE44-EsxV (HPE) trivalent vaccine, delivered as DNA, doubles IFN-γ levels versus BCG alone and elevates IL-12, IL-4, and TGF-β; BCG prime-boost amplifies cytokine responses (181). Liposomal HPE with DDA/TDB adjuvants enhances Th1 polarization and BCG immunological memory (182).

Further innovation yielded HPERC: HPE co-encapsulated with the TLR7/8 agonist resiquimod in size- and charge-optimized particles. HPERC improves APC uptake efficiency and Th1 activation in mice, marked by elevated IFN-γ. As a BCG booster, it augments IFN-γ, IL-17, and IgG2a—suggesting combined Th1/Th17 immunity (183).

### 8.4 PPE57

PPE57 displays strong immunogenicity but is encoded in the RD11 region, absent in BCG strains (203). The A3 vaccine—a fusion of Ag85B and PPE57—exemplifies multistage vaccine design. Delivered via plasmid DNA, protein, or lentiviral vectors, A3 induces Th1-polarized immunity in mice, activating CD4+/CD8+ T-cells, IFN-γ/TNF-α production, and antigen-specific antibodies (184, 185). In BCG prime-boost regimens, A3 reduces lung/spleen bacterial loads and lesion severity (186). Its successor, A39 (adding latency antigen Rv2029c), broadens protection to acute and latent TB, inhibiting bacterial reactivation and inflammation in pre- and post-exposure murine models (187).

The tetravalent Tetrafu56 vaccine (EspC, TB10.4, PPE57, HspX) targets replicative and dormant Mtb phases (188). While lacking prophylactic efficacy, it triggers robust IFN-γ in PBMCs from active TB patients—unlike healthy controls—suggesting therapeutic potential for Mtb-exposed individuals (162, 188). Notably, PPE57 exhibits structural variability across clinical strains, warranting further evaluation of population-level efficacy (73).

### 8.5 PPE68

The P9 peptide (aa 21–145) within PPE68’s RD1 region elicits IFN-γ production in PBMCs from TB patients and BCG-vaccinated individuals (73, 204). PPE68-expressing BCG (PPE68-rBCG) enhances Th1 responses without elevating virulence (189). The multivalent rLmMtb9Ag vaccine, which express nine immunoprotective Mtb antigens including PPE68 and is delivered via *Listeria monocytogenes*, activates CD4+/CD8+ T-cell proliferation and reduces lung/spleen bacterial loads in murine and guinea pig models. However, its protection against the Erdman strain remains inferior to BCG (190).

### 8.6 PPE15

PPE15, notable for conserved epitopes and roles in Mtb dormancy entry, is a vaccine candidate (73, 78, 97). Intranasal ChAdOx1.PPE15 boosts BCG efficacy in mice by amplifying CD4+/CD8+ T-cell responses and accelerating lung bacterial clearance (191).

### 8.7 PPE14

The rMTB41/AS02A subunit vaccine (PPE14-based) matches BCG’s protection in mice and guinea pigs. Immunized mice show Th1-polarized CD4+ and CD8+ T-cell responses with reduced lung CFUs; 60% of vaccinated guinea pigs survive 50 weeks post-challenge, contrasting with complete mortality in controls by 20 weeks (192).

### 8.8 Other PE/PPEs

Several additional PE/PPE proteins show immunomodulatory potential. The PPE39 homolog MTBK_24820 from Mtb Beijing/K drives Th1/IL-17 immunity in mice, matching BCG’s short-term protection but showing superior durability at 10 weeks post-infection (205). PE4 triggers IL-2/TNF/IL-6 production in mice, outperforming BCG in reducing lung CFUs and pathology by 45 days post-challenge (206). PPE26 promotes Th1 responses (elevated IFN-γ/IL-2) but lacks conservation and fails to protect mice despite inducing effector memory T-cells (207).

PE13, regulated by Rv0485 (which also controls PPE18), is conserved across lineages and elicits stronger T-cell responses in Mtb controllers versus progressors, suggesting protective potential (12, 208). PPE27, co-localized with PPE26 in the esx-5 locus, enhances *M.smegmatis* survival in murine tissues (124). PPE36-flagellin fusion induces Th1 immunity and splenocyte proliferation in mice (209). PE27 stimulates IFN-γ and memory T-cells in infected mice, though antigen specificity remains unconfirmed (146).

### 8.9 Challenges in targeting PE/PPE proteins for vaccination

Notably, the use of PE/PPE proteins as vaccine antigens also presents conceptual and practical challenges. As known immunomodulators, PE/PPE proteins can influence both innate and adaptive immune responses, raising the possibility that vaccines based on these proteins might dysregulate immunity rather than induce protective responses. Compounding this concern, *pe/ppe* genes in clinical Mtb isolates exhibit high genetic variability, potentially leading to antigenic divergence that may compromise vaccine efficacy across genetically diverse strain populations (65). Moreover, expression of these proteins is temporally and spatially dynamic, varying with infection stage and host environment—potentially limiting consistent antigen presentation during infection.

Future studies should address whether individual PE/PPE proteins in clinical isolates undergo antigenic variation, are differentially expressed, or are lost altogether. In parallel, detailed characterization of expression kinetics, antigen abundance, and cross-reactive immune responses is essential to evaluate their suitability as vaccine candidates.

### 8.10 Broader considerations in TB vaccine antigen selection

Clinical trial failures underscore the need to refine antigen selection criteria. Historically, immunodominant T-cell antigens—identified using PBMCs from active TB patients—guided target prioritization. This approach enabled Dillon et al. to isolate PPE18, leading to the M72/AS01E vaccine’s phase IIb success (166, 210). However, conserved immunodominant epitopes may benefit Mtb by diverting immune responses toward non-protective “decoys” (21, 211). Supporting this, weakly immunogenic antigens can confer stronger protection than dominant ones when adjuvanted (212), and most TB patient T-cell responses lack disease-modulating effects (12). Thus, immunodominance alone is an insufficient selection metric.

Further challenges arise from TB’s pulmonary tropism. Lung-resident memory T-cells (Trm), critical for local pathogen control, are underrepresented in peripheral blood analyses (213). Preclinical models must therefore incorporate lung-specific immunity assessments. Consensus favors tiered efficacy evaluation across mice, guinea pigs/rabbits, and nonhuman primates to improve translatability (198). Additionally, while prophylactic vaccines dominate research, therapeutic candidates—particularly for multidrug-resistant TB—demand exploration to shorten treatments and reduce relapse (214).

## 9 Diagnostic potential of PE/PPE family proteins

PE/PPE proteins show promise as serological or cellular biomarkers for tuberculosis. Several members stimulate Mtb-specific antibodies or cytokine profiles in PBMCs, distinguishing latent and active infections. PPE2 (Rv0256c), overexpressed during macrophage infection, outperforms PPD and ESAT-6 in detecting active TB across clinical subtypes (215). PPE17 demonstrates superior sensitivity for smear-negative TB and better distinguishes active TB from BCG-vaccinated individuals than PPE2 (216, 217). PPE42’s C-terminal MPTR sequence improves sensitivity in sputum/culture-negative cases, particularly when fused with other antigens (218). PE11, a virulence factor, elicits stage-specific antibodies; its co-transcription with PPE17 suggests complex formation, though diagnostic utility remains unexplored (219, 220).

PE35 and PPE68 trigger IFN-γ/IL-2 responses that differentiate active (IFN-γ-dominant) and latent (IL-2/dual-positive) TB via effector vs. memory T-cell profiles (11, 221). PPE41, particularly when complexed with PE25, boosts diagnostic sensitivity to 75% via enhanced B-cell responses (161). PE32-PPE65 induces Th2 responses that may compromise protection but could serve as diagnostic markers (152). PPE36-specific IgA (absent IgG) may offer TB-specific serological signatures (222). Recombinant PPE57 elicits IgG levels surpassing ESAT-6 and matching CFP-10, distinguishing TB patients from BCG-vaccinated controls (223).

## 10 Concluding remarks

The Mtb PE/PPE protein family, occupying ~10% of its genome, evolved from ancestral PE35-PPE68 pairs into diverse subfamilies through co-evolution with ESX secretion systems (7). These proteins form heterodimers or EspG-bound trimers for transport to the cell surface or secretion, with ESX-5 specifically exporting virulence-linked PE_PGRS and PPE_MPTR members (11, 61, 224). PE/PPE proteins mediate diverse roles in bacterial physiology and host-pathogen interactions, yet their evolutionary drivers—particularly the selective pressures driving their expansion and epitope conservation—remain enigmatic.

Structural and functional insights remain limited. While solved PE/PPE-EspG complexes reveal organizational principles (37, 225), most structures and proposed porin-like roles lack direct evidence. Similarly, their hypothesized role as ESX substrate channels awaits validation. Technical hurdles—including heterologous expression artifacts and functional redundancy—necessitate innovative approaches to study collective PE/PPE actions during infection.

Despite challenges, PE/PPE proteins offer untapped potential for TB interventions. Their conserved, cross-reactive T-cell epitopes underpin vaccine candidates like M72/AS01E, while clinical trial failures underscore the need to refine antigen selection beyond immunodominance. Therapeutic vaccines, critical for curbing drug-resistant TB, warrant urgent exploration.

Future research must bridge evolutionary, structural, and immunological gaps to unravel PE/PPE contributions to Mtb’s success and leverage these insights for next-generation diagnostics and vaccines.
